# Supplementary material for: Novel Driver Strength Index highlights important cancer genes in TCGA PanCanAtlas patients
Source: PeerJ. 2022 Aug 11;10:e13860. doi: 10.7717/peerj.13860 (PMC9375969; doi:10.7717/peerj.13860)
Supplement: Supplemental Information 2 [file peerj-10-13860-s002.zip › COHORTS/genes plots/cohorts/2021_8_16_14_9_distribution_cohorts_READ.pdf]

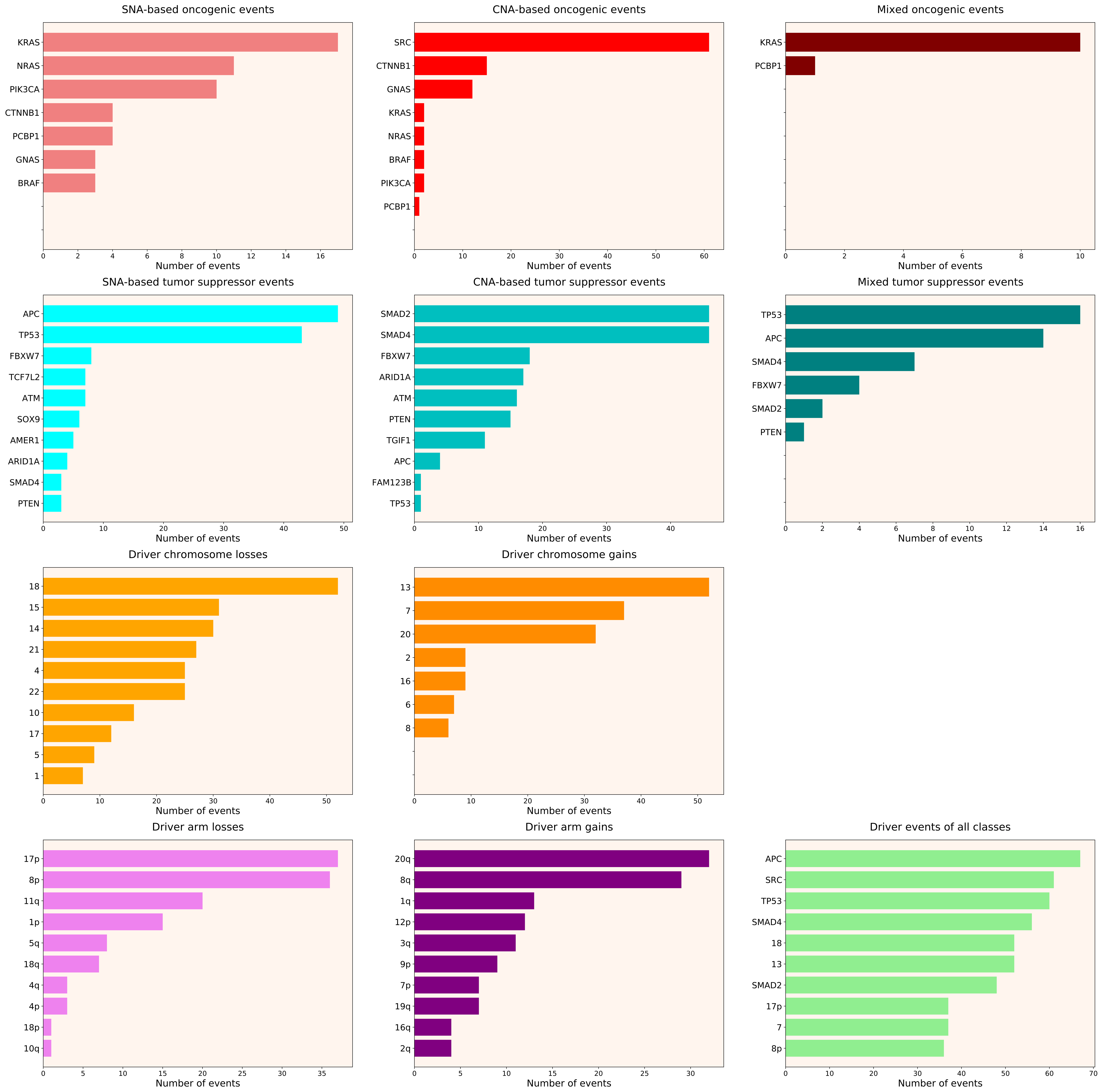

SNA-based oncogenic events
KRAS
NRAS
PIK3CA
CTNNB1
PCBP1
GNAS
BRAF
0 2 4 6 8 10 12 14 16
Number of events
CNA-based oncogenic events
SRC
CTNNB1
GNAS
KRAS
NRAS
BRAF
PIK3CA
PCBP1
0 10 20 30 40 50 60
Number of events
Mixed oncogenic events
KRAS
PCBP1
0 2 4 6 8 10
Number of events
SNA-based tumor suppressor events
APC
TP53
FBXW7
TCF7L2
ATM
SOX9
AMER1
ARID1A
SMAD4
PTEN
0 10 20 30 40 50
Number of events
CNA-based tumor suppressor events
SMAD2
SMAD4
FBXW7
ARID1A
ATM
PTEN
TGIF1
APC
FAM123B
TP53
0 10 20 30 40
Number of events
Mixed tumor suppressor events
TP53
APC
SMAD4
FBXW7
SMAD2
PTEN
0 2 4 6 8 10 12 14 16
Number of events
Driver chromosome losses
18
15
14
21
4
22
10
17
5
1
0 10 20 30 40 50
Number of events
Driver chromosome gains
13
7
20
2
16
6
8
0 10 20 30 40 50
Number of events
Driver arm losses
17p
8p
11q
1p
5q
18q
4q
4p
18p
10q
0 5 10 15 20 25 30 35
Number of events
Driver arm gains
20q
8q
1q
12p
3q
9p
7p
19q
16q
2q
0 5 10 15 20 25 30
Number of events
Driver events of all classes
APC
SRC
TP53
SMAD4
18
13
SMAD2
17p
7
8p
0 10 20 30 40 50 60 70
Number of events
